# The Current Status of Utilizing a Medication Record Handbook for Evaluating Shared Medication History: A Retrospective Study Using the Japanese National Claims Database

**DOI:** 10.7759/cureus.59096

**Published:** 2024-04-26

**Authors:** Hiroyuki Ura, Makoto Senoo, Kiyoshi Kubota, Kiyomi Sadamoto

**Affiliations:** 1 Department of Clinical Pharmacy, Shonan University of Medical Sciences, Yokohama, JPN; 2 Department of Pharmacy Services, Koshinkai Shiomidai Hospital, Yokohama, JPN

**Keywords:** retrospective study, covid-19 outbreak, patient-carried medication record handbook, external prescription rate, ndb open data japan

## Abstract

While the coronavirus disease 2019 (COVID-19) pandemic has impacted medication adherence and consultation patterns, its effects on the medical practice and dispensary separation system of Japan remain unclear. Thus, the utilization of the medication record handbook (MRH) in both medical and dental areas remains uncertain. This study uses the National Database of Health Insurance Claims and Specific Health Checkups of Japan (NDB); we analyzed the separation of medication prescription and dispensing in both medicine and dentistry, as well as estimated how much drug information is shared by utilizing a patient-carried MRH. The external prescription (EP) rate was used as the main indicator. We then analyzed the MRH utilization rate during outpatient medication guidance. During the pandemic, there was no distinctive change in the rate of EPs in both medicine and dentistry. Furthermore, an analysis between EPs, medical internal prescriptions (IPs), and dental IPs relative to the MRH utilization rate revealed significant correlations between EPs and medical IPs as well as medical and dental IPs. Conversely, no significant correlation was found between EPs and dental IPs. Therefore, our results suggest that active MRH implementation within healthcare facilities may lead to an increase in its utilization in dentistry.

## Introduction

The National Database of Health Insurance Claims and Specific Health Checkups of Japan (NDB) has operated under the Ministry of Health, Labor, and Welfare (MHLW) since 2009; it has been extensively used for research purposes since 2011 [1]. The NDB Open Data Japan consists of extremely high data coverage, including 98.3% of medical claims from hospitals and clinics, 97.8% of dental claims, and 99.7% of pharmacy prescriptions as of March 2022 [2]. It is a highly comprehensive and confidential healthcare database that remains exclusive to researchers with expertise in data security. Researchers are required to undergo an extended evaluation period before being granted direct access. This database was derived from the NDB and is made accessible to the public on an annual basis. It also includes information on medical, dental, and dispensing service fees per prefecture and five-year age groups. Therefore, researchers can analyze regional or age-group variations using NDB Open Data Japan in relation to their associated services.

In recent years, several studies have reported on the impact of the coronavirus disease 2019 (COVID-19) pandemic on medication adherence and consultation patterns. According to a recent report, there has been a decrease in hospital admissions for respiratory diseases, endoscopic procedures, and other systemic illnesses as well as pediatric outpatient visits and admissions in Japan immediately following the pandemic [3]. Osawa et al. reported that although there was a transient decrease in the number of physician visits immediately post-pandemic, there was no change in the proportion of days covered by medication prescription [4].

Previous reports revealed that Frederick II of the Holy Roman Empire instituted the separation of medication prescription and dispensing to prevent poisoning. He separated those who examined the sick and wrote death certificates (physicians) from those who rigorously administered medications (pharmacists) [5]. Today, this system has become widespread, wherein the responsibilities of pharmacists and physicians are carefully separated. Two primary advantages were associated with this system. First, physicians and pharmacists can ensure patient safety by performing their respective professional duties. The pharmacist checks the prescription, assesses the patient's medication history, examines medication errors, and offers advice on how to avoid adverse effects. Second, medication overdoses were mitigated. In 2020, Japan had a drug expenditure/GDP ratio of 1.99%, which was comparatively elevated compared to other Western nations [6]. Thus, pharmacists should observe appropriate drug usage by avoiding duplicate dosing, discontinuing unnecessary prescriptions, and/or suggesting a reduction in its use.

Although the separation system may have its benefits, disadvantages were noted when understanding how prescriptions have been given at other medical institutions. In 1994, a drug-related accident occurred in Japan; solivudine, a medication against viral herpes, was combined with 5-fluorouracil, an anti-cancer agent. This error led to the unfortunate demise of 18 individuals as well as multiple instances of serious adverse effects such as hematopoietic injury [7,8]. The accident prompted the development of the patient-carried medication record handbook (MRH), now known as "Okusuri-techo." This system facilitates the sharing of prescription information among healthcare facilities, thereby decreasing the risk of drug interactions and dose duplications. The patient-carried MRH, which allowed outpatients to conveniently access their medication information, acquired significant recognition after certain disasters, i.e., the Great Hanshin-Awaji Earthquake in 1995 and the subsequent Great East Japan Earthquake in 2011 [9]. Therefore, adopting this system is being actively promoted as a national policy. However, large-scale data analyses indicating its usage extent for medication guidance remain unclear.

This study aimed to analyze the actual situation of the separation system for medication prescription and dispensing in both medicine and dentistry by using NDB Open Data Japan, as well as to determine the MRH utilization rate when providing medication guidance to outpatients.

## Materials and methods

Data source

In this study, records from the 3rd to 8th NDB Open Data Japan within a six-year span (Japanese fiscal year FY 2016 to FY 2021 ,i.e., April 1, 2016 to March 31, 2022) were downloaded from the MHLW website [10]. Records were then categorized according to sex, five-year age groups, and 47 prefectures in Japan and were as follows: (a) medical and dental supervision fees, (b) dispensing fees to external or internal prescriptions (IPs or EPs) in both outpatient medical and dental practices, and (c) pharmaceutical management fees in the outpatient pharmaceutical dispensing services.

Ep rate

We utilized the EP rate as an indicator for the separation of medication prescription and dispensing in medicine and dentistry. It was calculated by dividing the number of issued EPs by the number of all prescriptions issued for outpatients. Table 1 shows the medical and dental practice codes used in this analysis.

**Table 1:** Practice code used to calculate the EP rate. EPs: external prescriptions; IPs: internal prescriptions

Category	Practice code
EPs	
Medical prescriptions issued	120002710
	120002610
	120003610
	120004410
Dental prescription issued	306001210
	306001310
IPs	
Medical prescription issued	120001210
	120002610
	120003610
	120004410
Dental prescription issued	306000610
	306000710

Utilization of MRH during outpatient medication guidance

We analyzed the MRH utilization rate during outpatient medication guidance as defined: (1) IPs, the ratio of cases with additional fees for including MRH utilization to cases with fees for providing medication information and guidance; (2) EPs, the ratio of calculations for implementing medication guidance using MRH to the number of accounts for the pharmacy management fee associated with its implementation. In this analysis, we utilized the medical, dental, and dispensing practice codes as shown in Table 2.

**Table 2:** Practice code used to calculate the utilization of MRH during outpatient medication guidance. EPs: external prescriptions; IPs: internal prescriptions; MRH: medication record handbook

Category	Practice code
EPs	
Medication guidance with MRH	440004010
	440004510
	440007810
	440008110
	440008910
Medication guidance without MRH	440007910
	440008210
	440009010
Unknown	440008010
	440008310
	440008410
	440009110
IPs	
Medication guidance in medical practice	113701310
Adding medication information to MRH in medical practice	120002370
Medication guidance in dental practice	302003470
Adding medication information to MRH in dental practice	302003310

Factors related to the MRH utilization rate during medication guidance

We examined the factors associated with the MRH utilization rate by using the median group age and prefecture area. The median age by prefecture was based on the latest results of the Japanese census, which is compiled every five years as of October 1, 2020 [11].

Data analysis

The data was collected using a standardized extraction form and subsequently recorded in an Excel spreadsheet. We used scatter plots and Spearman's correlation to show the association between EP rates in medicine and dentistry as well as the MRH utilization rate during outpatient medication guidance for EPs and medical/dental IPs. Graphs were created accordingly and statistical analysis was performed using GraphPad Prism 9 for Mac (GraphPad Software, Inc., USA). A P-value <0.05 was considered statistically significant.

Ethical consideration

This study relied on available literature and anonymized open data. No ethical concerns were of relevance to the current study.

## Results

External prescription rate

**Figure 1:**
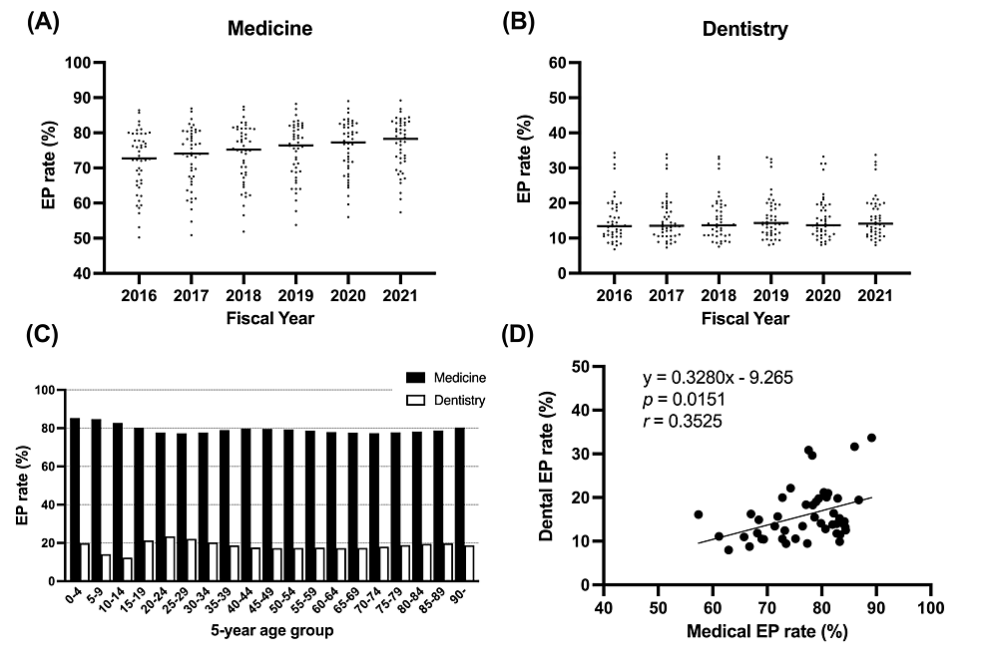
EP rate from FY 2016 until FY 2021. EP rate in the (A) medical and (B) dental services. Dot plots indicate the EP rate in each prefecture. Horizontal lines indicate median values for each fiscal year in Japan. (C) EP rate is categorized by age group at intervals of five years. (D) Correlations between EP rates in medical and dental practices. Dot plots indicate the EP rate in each prefecture. EP: external prescription

During the analysis period, the median medical EP rate calculated for each of the 47 prefectures increased from 72.7% (FY 2016) to 78.3% (FY 2021) (Figure 1A). In contrast, there was no notable change in the median dental EP rate, which remained at 13.4% (FY 2016), 13.5% (FY 2017), 14.3% (FY 2018), 13.7% (FY 2019), 13.7% (FY 2020), and 14.1% (FY 2021) (Figure 1B). In FY 2021, the highest medical EP rate was found in the Kanagawa prefecture (89.2%), while the lowest was in the Fukui prefecture (57.4%). The highest dental EP rate was also found in the Kanagawa prefecture (33.7%), while the lowest was in the Tokushima prefecture (8.0%). When analyzed using the five-year age group, no notable trends were observed in either the medical or dental EP rates; the dental EP rate declined during childhood, with the lowest (12.3%) among those between 10 and 14 years of age (Figure 1C). A weak correlation was also found between medical versus dental EP rates (r=0.3525, p=0.0151, Figure 1D).

Utilization of MRH during outpatient medication guidance

**Figure 2:**
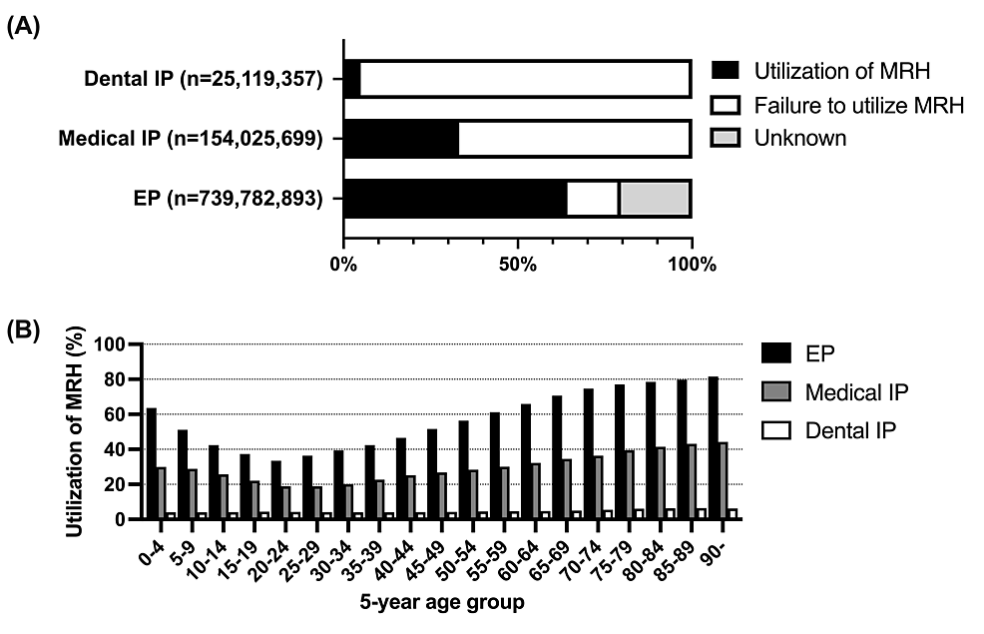
Utilization of MRH for medication guidance during FY 2021. EP data is shown as a hybrid of medical and dental prescribing. (A) Proportion of utilization of MRH for medication guidance. (B) Proportion of MRH utilization during medication guidance, categorized by age group at intervals of five years. EP: external prescription; MRH: medication record handbook

During outpatient medication guidance in FY 2021, MRH was utilized by 64.2% of EPs, 33.2% of medical IPs, and 4.9% of dental IPs (Figure 2A). Analysis by five-year age group showed that in the medical practice, both EPs and IPs were at their lowest point in the 20-year age group (EPs: 33.4% of those 20-24 years, IPs: 18.9% of those 25-29 years), with a gradual increase thereafter; 81.6% of those aged ≥90 years used MRH for EPs and 44.3% of those were for IPs in the medical practice (Figure 2B). Conversely, in the dental practice, there was no notable change; although, there was a slightly increasing trend among those aged ≥40 years, wherein 6.5% of the highest age group (85-89 years) utilized MRH for medication guidance (Figure 2B).

Factors related to the MRH utilization rate during medication guidance

**Figure 3:**
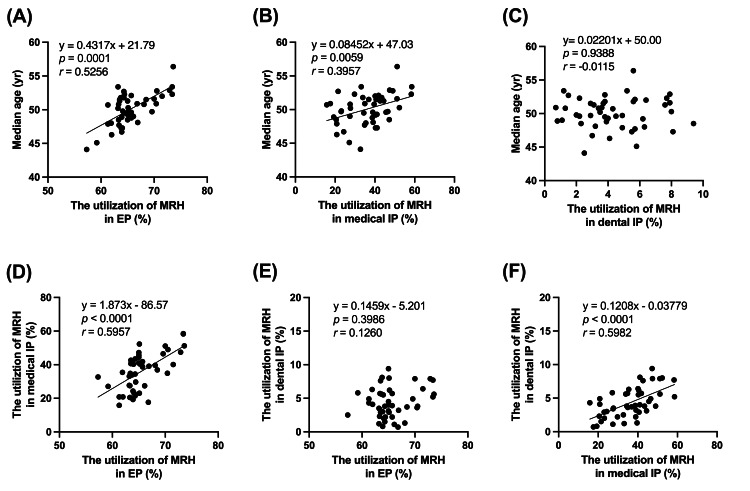
Scatter plots related to the utilization of MRH for medication guidance during FY 2021. Scatter plots illustrating average age and the utilization of MRH during medication guidance for (A) EP, (B) medical IP, and (C) dental IP. In addition, a scatter plot illustrating the utilization of MRH during medication guidance for (D) EP versus medical IP, (E) EP versus dental IP, and (F) medical versus dental IP. Dot plots indicate the median age or the percentage of the utilization of MRH in each prefecture. EP: external prescription; MRH: medication record handbook

There was a significant correlation between median age and the MRH utilization rate among the EPs (r=0.5256, p=0.0001; Figure 3A). Similarly, there was a moderately positive association between median age and the MRH utilization rate among the medical IPs (r=0.3957, p=0.0059; Figure 3B). No significant correlation was found between median age and the MRH utilization rate among the dental IPs (p=0.9388; Figure 3C).

Analysis between EPs, medical IPs, and dental IPs in relation to the MRH utilization rate showed significant correlations between EPs versus medical IPs (r=0.5857, p<0.0001; Figure 3D) and medical IPs versus dental IPs (r=0.5982, p<0.0001; Figure 3F). In contrast, no significant correlation was found between EPs versus dental IPs (p=0.3986, Figure 3E).

## Discussion

Our study investigated the present state of the separation system for medication prescription and dispensing by using the outpatient EP rate as the main indicator and evaluated the status of utilizing the MRH for sharing medication information. In this study, we revealed the trend of the separation system by using records within the NDB Open Data Japan from FY 2016 to FY 2021 before the MRH utilization survey (Figure 1). In this study, we used the EP rate as an indicator for the separation system and found no notable variations among the EP rates in the medical and dental fields between FY 2020 and FY 2021, despite the occurrence of the COVID-19 pandemic.

We also revealed that MRH utilization for medication guidance remains extremely minimal in the dental field (Figure 2). Additionally, NDB Open Data Japan does not provide a breakdown of medical or dental EPs. Consequently, the distribution between medical and dental EPs remains unclear. However, our investigation indicated that >60% of EPs were provided with MRH-assisted medication guidance (Figure 2A). However, since pharmacies provide medication guidance under the same system for both medical and dental outpatients, a notable difference in the MRH utilization rate between the medical and dental practices remains unlikely.

This study was the first to demonstrate that medication guidance utilizing MRH is significantly correlated with age for EPs or medical IPs, but not for dental IPs (Figures 3A-3C). Most prescriptions were made for medical outpatients (Figure 1B). In addition, the utilization rate of MRH at the time of medication guidance in dentistry remains extremely low (Figure 2A). Therefore, it can be concluded that the MRH utilization rate at the time of medication guidance tends to increase in an age-dependent manner among medical outpatients, whereas this remains age-independent and low among dental outpatients. The consolidation of medication histories using the MRH may be beneficial not only for collaboration between medical institutions and external pharmacies but also between dental practices and external pharmacies.

During dental treatment involving invasive procedures such as tooth extraction, osteoporosis drugs such as bisphosphonates, receptor activators of nuclear factor-kappa B ligand (RANKL) antibodies, and antithrombotic drugs may adversely affect treatment [12-14]. In addition, several central nervous system medications, such as antidepressants and antipsychotics, caused oral-related symptoms, including xerostomia, gingival proliferation, non-dental toothache, and temporomandibular joint pain. Furthermore, non-steroidal anti-inflammatory drugs (NSAIDs) and macrolide antibacterial drugs prescribed during dental visits may enhance the bioavailability of antiepileptic and antithrombotic drugs prescribed during medical consultations. Based on a comprehensive retrospective study using NDB data from 2010 to 2016, it was found that 16.5-17.9% of patients aged 65-79 years and 28.6-33.0% of patients aged ≥80 years were taking five or more oral medications [15]. Moreover, at least 15.9% of outpatients aged ≥65 years had a dental visit in Japan as of October 2020 [16]. Therefore, it is crucial to share prescription histories during medical and dental consultations in order to ensure treatment safety.

Research findings indicated a correlation between the MRH utilization rate among the medical and dental IPs (Figure 3F). The correlation between the MRH utilization rate for EPs and dental IPs was probably due to an age-dependent increase in the MRH utilization rate in both cases (Figure 3D). However, as the MRH utilization rate for dental IPs remained age-independent, its correlation between medical and dental IPs was probably due to factors other than age. The fact that there was a correlation between the MRH utilization rate among the medical and dental practices, even though there was no correlation between EPs and dental IPs, suggested that the active use of MRH in medical facilities was more likely to lead to higher MRH utilization in dentistry, rather than when being promoted by outpatient pharmacies. In a survey of 148 pharmacists from 73 pharmacies associated with the Otaru Pharmaceutical Association and of 157 patients purchasing from those dispensing pharmacies, approximately 80.9% of patients and 100% of pharmacists indicated that MRH was indeed beneficial [17]. A total of 93.6% of the patients who utilized the MRH stated that they mostly delivered their MRH when purchasing from dispensing pharmacies, while 36.9% did so at hospitals [17]. Therefore, it will be essential to promote the importance of presenting the MRH in hospitals and clinics in the future.

This study had some limitations. First, multivariate analyses were deemed complicated when using NDB Open Data Japan for this study. Our analysis of data based on the five-year age group and 47 prefectures indicated that age had little effect on EP rate but had a notable effect on the MRH utilization rate (Figures 1C, 2B). However, a comprehensive analysis of variables other than generation, such as comorbidities and the effect of multiple concomitant medications, may provide more valuable data for advancing the separation system for medication prescription and dispensing as well as the use of the MRH. It would be beneficial to conduct more investigations utilizing datasets that include databases that relay private sector receipts and NDB data. Second, the MRH utilization rate in our study was based on the calculated requirement for reimbursement, which is the presentation of MRH by the patient and the subsequent filling of prescription drug information. In some clinical practices, there may be cases where professionals check the MRH but do not claim reimbursement because of the time and effort required to provide the information. Therefore, the estimated rate of MRH utilization in this research may underestimate the number of cases in which MRH was only checked but not filled in with the appropriate information.

## Conclusions

In conclusion, we analyzed the current status of the separation system for medication prescription and dispensing in Japan as well as the sharing of medication information among health facilities by utilizing the patient-carried MRH. It was shown that the rates of separating medication prescriptions and dispensing and utilizing the MRH were lower in the dental field than in the medical field. Therefore, it may be considered essential to encourage the use of MRH to provide safe and effective pharmacotherapy, especially in older adults with multiple medications who may need more opportunities to regularly visit their dentists.
